# Conditional Deletion of All Neurexins Defines Diversity of Essential Synaptic Organizer Functions for Neurexins

**DOI:** 10.64898/2026.07.15.738748

**Published:** 2026-08-31

**Authors:** Man Jiang, Yang Li, Margarita Artyukhova, Li Zhang, Shu Luo, Weiran Zhan, Bo Zhang, Ozgun Gokce, Thomas C. Südhof

## Abstract

Presynaptic neurexins are key regulators of synapse properties that arguably represent the best-studied synaptic adhesion molecules. Despite thousands of papers, however, no direct comparison of overall neurexin functions in different types of synapses is available. A decade ago, we provided such an analysis but we recently retracted this paper because four images contained microduplications that, although without discernible impact on the paper’s conclusions, could not be corrected. As a result, the scientific community lost access to primary data that established the fundamental principle that neurexins perform profoundly different functions in different types of synapses. In the present study, we have therefore reanalyzed the original raw data and expanded their conclusions with new experiments to document, in a single study, the basic contributions of neurexins to different synapses. We used triple conditional knockout mice targeting all neurexins except for Neurexin-1γ and applied neuron-specific manipulations combined with slice electrophysiology, two-photon Ca ^2+^ -imaging, and immunohistochemistry. With this approach, we focused on excitatory climbing-fiber synapses in the cerebellum and on inhibitory synapses formed by parvalbumin- or somatostatin-positive neurons in the cerebellum, hippocampus, and medial prefrontal cortex. Our results show that pan-neurexin deletions produce dramatically different phenotypes in synapses, ranging from modest to massive impairments in synapse assembly (climbing-fiber and parvalbumin-positive synapses) to severe but selective decreases in action potential-induced presynaptic Ca ^2+^ -transients (somatostatin-positive synapses). Thus, neurexins perform powerful but distinct context-dependent roles in different synapses that shape the brain’s circuits.

**Significance Statement:**

Neurexins are abundant presynaptic adhesion molecules expressed from three genes in thousands of splice variants. Although neurexins are well studied, no analysis that compares neurexin deletions in multiple types of synapses is currently available. Here, we provide such an analysis by examining triple conditional knockout mice that delete all neurexins except for Neurexin-1γ. Using neuron-specific manipulations combined with slice electrophysiology, two-photon Ca ^2+^ imaging and immunohistochemistry, we show that pan-neurexin deletions produce distinct phenotypes in different synapses, ranging from impairments in synapse assembly (climbing-fiber and parvalbumin-positive cortical synapses) to severe selective decreases in presynaptic action potential-induced Ca ^2+^ -transients (somatostatin-positive cortical synapses). Our data reveal context-dependent distinct functions of neurexins in different synapses whose properties shape the input-output relations of neural circuits.

## Full Text Availability

The license terms selected by the author(s) for this preprint version do not permit archiving in PMC. The full text is available from the preprint server.

